# Tri-(2-ethylhexyl)trimellitat – Bestimmung von 1-MEHTM, 2-MEHTM, 5OH-1-MEHTM, 5OH-2-MEHTM, 5cx-1-MEPTM und 5cx-2-MEPTM in Urin mittels LC-MS/MS: Biomonitoring-Methode

**DOI:** 10.34865/bi331931d10_4or

**Published:** 2025-12-22

**Authors:** Laura Kuhlmann, Elisabeth Eckert, Christine Höllerer-Mittelmaier, Thomas Göen, Veronika Spindler, Gerhard Scherer, Craig Sams, Kate Jones, Andrea Hartwig, MAK Commission

**Affiliations:** 1 Friedrich-Alexander-Universität Erlangen-Nürnberg. Institut und Poliklinik für Arbeits-, Sozial- und Umweltmedizin Henkestraße 9–11 91054 Erlangen Deutschland; 2 ABF Analytisch-biologisches Forschungslabor GmbH Semmelweisstraße 5 82152 Planegg Deutschland; 3 Health and Safety Executive (HSE) Science and Research Centre Harpur Hill SK17 9JN Buxton (Derbyshire) Vereinigtes Königreich; 4 Institut für Angewandte Biowissenschaften. Abteilung Lebensmittelchemie und Toxikologie. Karlsruher Institut für Technologie (KIT) Adenauerring 20a, Geb. 50.41 76131 Karlsruhe Deutschland; 5 Ständige Senatskommission zur Prüfung gesundheitsschädlicher Arbeitsstoffe. Deutsche Forschungsgemeinschaft, Kennedyallee 40, 53175 Bonn, Deutschland. Weitere Informationen: Ständige Senatskommission zur Prüfung gesundheitsschädlicher Arbeitsstoffe | DFG

**Keywords:** TEHTM, Biomonitoring, Urin, LC-MS/MS

## Abstract

The working group “Analyses in Biological Materials” of the German Senate Commission for the Investigation of Health Hazards of Chemical Compounds in the Work Area (MAK Commission) developed and verified this biomonitoring method for the measurement of six specific metabolites of the plasticiser tri‑(2‑ethylhexyl) trimellitate (TEHTM) in urine. Specifically, this method determines two monoester isomers as primary hydrolysis products of TEHTM, 1‑mono-(2‑ethylhexyl) trimellitate (1‑MEHTM) and 2‑mono-(2‑ethylhexyl) trimellitate (2‑MEHTM), as well as the oxidatively formed secondary derivatives, namely 1‑mono-(2‑ethyl-5‑hydroxyhexyl) trimellitate (5OH‑1‑MEHTM), 2‑mono-(2‑ethyl-5‑hydroxyhexyl) trimellitate (5OH‑2‑MEHTM), 1‑mono-(2‑ethyl-5‑carboxypentyl) trimellitate (5cx‑1‑MEPTM), and 2‑mono-(2‑ethyl-5‑carboxypentyl) trimellitate (5cx‑2‑MEPTM). Determination is carried out after enzymatic hydrolysis of the urine sample as well as enrichment of the analytes by online SPE. Via integrated, automatic column-switching, the analytes are transferred onto the analytical column in backflush mode, separated by liquid chromatography, and quantified by tandem mass spectrometry. Calibration is performed using calibration standards prepared in pooled urine and processed analogously to the samples to be analysed. The following isotope-labelled substances are added to the urine samples as internal standards: D_5_‑1‑MEHTM, D_5_‑2‑MEHTM, D_5_‑5OH‑1‑MEHTM, D_5_‑5cx‑1‑MEPTM, and D_5_‑5cx‑2‑MEPTM. The method provides reliable and accurate analytical results, as shown by the good precision data with standard deviations no greater than 8%. Good accuracy data were obtained with mean relative recoveries in the range of 97–109%. The method is both selective and sensitive, and provides quantitation limits in the range of 0.04–0.12 μg/l.

## 1 Kenndaten der Methode

**Table:** 

**Matrix**	Urin		
**Analytisches Messprinzip**	Flüssigkeitschromatographie mit Tandem-Massenspektrometrie (LC‑MS/MS)		
**Parameter und entsprechender Arbeitsstoff**			
**Arbeitsstoff**	**CAS‑Nr.**	**Parameter**	**CAS‑Nr.**
Tri-(2‑ethylhexyl)trimellitat (TEHTM)	3319-31-1	1‑Mono-(2‑ethylhexyl)trimellitat (1‑MEHTM)	61137-09-5
		2‑Mono-(2‑ethylhexyl)trimellitat (2‑MEHTM)	63468-08-6
		1‑Mono-(2‑ethyl-5‑hydroxyhexyl)trimellitat (5OH‑1‑MEHTM)	2306733-44-6
		2‑Mono-(2‑ethyl-5‑hydroxyhexyl)trimellitat (5OH‑2‑MEHTM)	2306733-45-7
		1‑Mono-(2‑ethyl-5‑carboxypentyl)trimellitat (5cx‑1‑MEPTM)	2306733-48-0
		2‑Mono-(2‑ethyl-5‑carboxypentyl)trimellitat (5cx‑2‑MEPTM)	2306733-49-1

### Zuverlässigkeitskriterien

#### 1‑MEHTM

**Table:** 

Präzision in der Serie:	Standardabweichung (rel.)	*s*_w_ = 3,0 % bzw. 6,3 %
	Streubereich	*u* = 6,8 % bzw. 14,3 %
	bei einer dotierten Konzentration von 0,24 μg oder 1,20 μg 1‑MEHTM pro Liter Urin und n = 10 Bestimmungen	
Präzision von Tag zu Tag:	Standardabweichung (rel.)	*s*_w_ = 5,2 % bzw. 6,6 %
	Streubereich	*u* = 12,3 % bzw. 15,9 %
	bei einer dotierten Konzentration von 0,24 μg oder 1,20 μg 1‑MEHTM pro Liter Urin und n = 8 Bestimmungen	
Richtigkeit:	Wiederfindung (rel.)	*r* = 100 % bzw. 99,0 %
	bei einer dotierten Konzentration von 0,24 μg oder 1,20 μg 1‑MEHTM pro Liter Urin und n = 8 Bestimmungen	
Nachweisgrenze:	0,01 μg 1‑MEHTM pro Liter Urin	
Bestimmungsgrenze:	0,04 μg 1‑MEHTM pro Liter Urin	

#### 2‑MEHTM

**Table:** 

Präzision in der Serie:	Standardabweichung (rel.)	*s*_w_ = 4,3 % bzw. 6,3 %
	Streubereich	*u* = 9,7 % bzw. 14,3 %
	bei einer dotierten Konzentration von 0,25 μg oder 1,27 μg 2‑MEHTM pro Liter Urin und n = 10 Bestimmungen	
Präzision von Tag zu Tag:	Standardabweichung (rel.)	*s*_w_ = 4,0 % bzw. 7,3 %
	Streubereich	*u* = 9,5 % bzw. 17,3 %
	bei einer dotierten Konzentration von 0,25 μg oder 1,27 μg 2‑MEHTM pro Liter Urin und n = 8 Bestimmungen	
Richtigkeit:	Wiederfindung (rel.)	*r* = 104 % bzw. 96,8 %
	bei einer dotierten Konzentration von 0,25 μg oder 1,27 μg 2‑MEHTM pro Liter Urin und n = 8 Bestimmungen	
Nachweisgrenze:	0,02 μg 2‑MEHTM pro Liter Urin	
Bestimmungsgrenze:	0,07 μg 2‑MEHTM pro Liter Urin	

#### 5OH‑1‑MEHTM

**Table:** 

Präzision in der Serie:	Standardabweichung (rel.)	*s*_w_ = 5,0 % bzw. 4,0 %
	Streubereich	*u* = 11,3 % bzw. 9,0 %
	bei einer dotierten Konzentration von 0,26 μg oder 1,28 μg 5OH‑1‑MEHTM pro Liter Urin und n = 10 Bestimmungen	
Präzision von Tag zu Tag:	Standardabweichung (rel.)	*s*_w_ = 5,0 % bzw. 3,7 %
	Streubereich	*u* = 11,8 % bzw. 8,8 %
	bei einer dotierten Konzentration von 0,26 μg oder 1,28 μg 5OH‑1‑MEHTM pro Liter Urin und n = 8 Bestimmungen	
Richtigkeit:	Wiederfindung (rel.)	*r* = 98,9 % bzw. 99,0 %
	bei einer dotierten Konzentration von 0,26 μg oder 1,28 μg 5OH‑1‑MEHTM pro Liter Urin und n = 8 Bestimmungen	
Nachweisgrenze:	0,02 μg 5OH‑1‑MEHTM pro Liter Urin	
Bestimmungsgrenze:	0,07 μg 5OH‑1‑MEHTM pro Liter Urin	

#### 5OH‑2‑MEHTM

**Table:** 

Präzision in der Serie:	Standardabweichung (rel.)	*s*_w_ = 8,1 % bzw. 5,5 %
	Streubereich	*u* = 18,3 % bzw. 12,4 %
	bei einer dotierten Konzentration von 0,25 μg oder 1,24 μg 5OH‑2‑MEHTM pro Liter Urin und n = 10 Bestimmungen	
Präzision von Tag zu Tag:	Standardabweichung (rel.)	*s*_w_ = 5,9 % bzw. 2,7 %
	Streubereich	*u* = 14,0 % bzw. 6,3 %
	bei einer dotierten Konzentration von 0,25 μg oder 1,24 μg 5OH‑2‑MEHTM pro Liter Urin und n = 8 Bestimmungen	
Richtigkeit:	Wiederfindung (rel.)	*r* = 104 % bzw. 109 %
	bei einer dotierten Konzentration von 0,25 μg oder 1,24 μg 5OH‑2‑MEHTM pro Liter Urin und n = 8 Bestimmungen	
Nachweisgrenze:	0,04 μg 5OH‑2‑MEHTM pro Liter Urin	
Bestimmungsgrenze:	0,12 μg 5OH‑2‑MEHTM pro Liter Urin	

#### 5cx‑1‑MEPTM

**Table:** 

Präzision in der Serie:	Standardabweichung (rel.)	*s*_w_ = 2,4 % bzw. 6,0 %
	Streubereich	*u* = 5,4 % bzw. 13,6 %
	bei einer dotierten Konzentration von 0,24 μg oder 1,20 μg 5cx‑1‑MEPTM pro Liter Urin und n = 10 Bestimmungen	
Präzision von Tag zu Tag:	Standardabweichung (rel.)	*s*_w_ = 6,0 % bzw. 4,8 %
	Streubereich	*u* = 14,2 % bzw. 11,4 %
	bei einer dotierten Konzentration von 0,24 μg oder 1,20 μg 5cx‑1‑MEPTM pro Liter Urin und n = 8 Bestimmungen	
Richtigkeit:	Wiederfindung (rel.)	*r* = 105 % bzw. 109 %
	bei einer dotierten Konzentration von 0,24 μg oder 1,20 μg 5cx‑1‑MEPTM pro Liter Urin und n = 8 Bestimmungen	
Nachweisgrenze:	0,01 μg 5cx‑1‑MEPTM pro Liter Urin	
Bestimmungsgrenze:	0,05 μg 5cx‑1‑MEPTM pro Liter Urin	

#### 5cx‑2‑MEPTM

**Table:** 

Präzision in der Serie:	Standardabweichung (rel.)	*s*_w_ = 4,8 % bzw. 6,0 %
	Streubereich	*u* = 10,9 % bzw. 13,6 %
	bei einer dotierten Konzentration von 0,24 μg bzw. 1,21 μg 5cx‑2‑MEPTM pro Liter Urin und n = 10 Bestimmungen	
Präzision von Tag zu Tag:	Standardabweichung (rel.)	*s*_w_ = 4,8 % bzw. 5,6 %
	Streubereich	*u* = 11,4 % bzw. 13,2 %
	bei einer dotierten Konzentration von 0,24 μg bzw. 1,21 μg 5cx‑2‑MEPTM pro Liter Urin und n = 8 Bestimmungen	
Richtigkeit:	Wiederfindung (rel.)	*r* = 99,4 % bzw. 100 %
	bei einer dotierten Konzentration von 0,24 μg bzw. 1,21 μg 5cx‑2‑MEPTM pro Liter Urin und n = 8 Bestimmungen	
Nachweisgrenze:	0,01 μg 5cx‑2‑MEPTM pro Liter Urin	
Bestimmungsgrenze:	0,04 μg 5cx‑2‑MEPTM pro Liter Urin	

## 2 Allgemeine Informationen zu TEHTM

Tri‑(2‑ethylhexyl)trimellitat (TEHTM) ist ein Weichmacher der insbesondere als Alternativprodukt für Di‑(2‑ethylhexyl)­­phthalat (DEHP) verwendet wird (Bourdeaux et al. 2016; SCENIHR 2015; Van Vliet et al. 2011). TEHTM zeichnet sich im direkten Vergleich zu DEHP sowohl durch eine geringere Toxizität als auch durch eine deutlich geringere Migrationsrate in Kontaktmedien aus (Eckert et al. 2016). TEHTM wird bisher vorrangig für Medizinprodukte aus Weich‑PVC verwendet, insbesondere für Beutel und Schläuche, die für Blut, Plasma, Infusionen oder künstliche Ernährung bestimmt sind. Der Anteil der Weichmacher im PVC‑Material beträgt dabei üblicherweise 20–40 % des Gesamtgewichts (Bernard et al. 2014; Green et al. 2005). Da die Weichmacher nicht chemisch an das PVC gebunden sind, können diese in das Kontaktmedium, z. B. Blut, migrieren. Dadurch kann es zu einer direkten Exposition von Patienten kommen, was auch die wichtigste Expositionsquelle für TEHTM darstellt. Bereits vorhandene Zell- und Tierstudien zu TEHTM wurden meist in direktem Vergleich mit DEHP durchgeführt. Hierbei konnte eine deutlich geringere Toxizität von TEHTM im Vergleich zu DEHP festgestellt werden (Eljezi et al. 2017; Hodgson 1987; Kambia et al. 2004; Ohashi et al. 2005). Da keine Humandaten vorlagen, wurde im Rahmen des BMU‑VCI-Projekts in einer In-vivo-Studie der Humanmetabolismus von TEHTM erstmalig untersucht (Höllerer et al. 2018 a). Die hierzu angewandte Methode wurde international publiziert (Höllerer et al. 2018 b).

**Abb.1:**
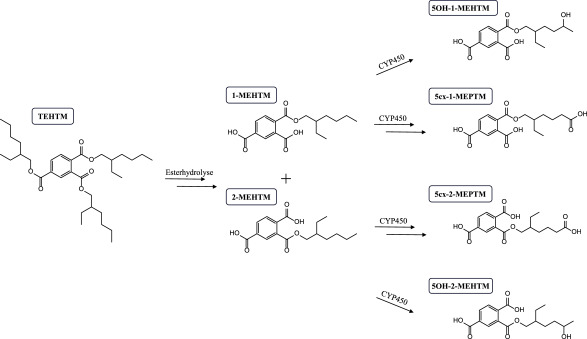
Metabolismusschema von TEHTM, stark vereinfacht, nach Höllerer et al. (2018 a) und Kuhlmann et al. (2021)

Die Ergebnisse der In-vivo-Studie zeigen, dass TEHTM nach oraler Aufnahme resorbiert und zunächst selektiv zu den Diester-Isomeren 1,2‑Di‑(2‑ethylhexyl)trimellitat (1,2‑DEHTM) und 2,4‑Di‑(2‑ethylhexyl)trimellitat (2,4‑DEHTM) gespalten wird. Diese werden dann weiter zu den Monoester-Isomeren 1‑MEHTM und 2‑MEHTM abgebaut. Das Diester-Isomer 1,4‑Di‑(2‑ethylhexyl)trimellitat (1,4‑DEHTM) sowie der Monoester 4‑MEHTM konnten hingegen im Blut nicht nachgewiesen werden. Im Urin konnten alle drei Monoester-Isomere, 1‑MEHTM, 2‑MEHTM und 4‑MEHTM, nachgewiesen werden, wobei 4‑MEHTM nur in sehr geringen Mengen gefunden wurde. Für die relevanten Monoester 1‑MEHTM und 2‑MEHTM wurden auch sekundäre Metaboliten (Oxidation an der Seitenkette) untersucht und konnten im Urin der exponierten Personen ebenfalls nachgewiesen werden. Basierend auf diesen Ergebnissen konnte ein Metabolismusschema von TEHTM aufgestellt werden, welches in Abbildung 1 dargestellt ist. Generell zeigte sich, dass die Metabolisierung von TEHTM und die Ausscheidung der Metaboliten mit dem Urin beim Menschen relativ langsam verläuft, da einige Metaboliten auch noch 72 h nach Exposition im Urin nachweisbar waren (Höllerer et al. 2018 a). Bei fünf der mit dieser Methode erfassbaren Metaboliten wurde eine zweiphasige Eliminationskinetik beobachtet. Dabei wurden für die Exkretion mit dem Urin Halbwertszeiten zwischen 4 und 6 Stunden bzw. zwischen 10 und 33 Stunden berechnet (Höllerer et al. 2018 a). Für 5cx‑2‑MEPTM wurde eine einphasige Eliminationskinetik mit einer Halbwertszeit von 17 h beschrieben. Insgesamt wurden innerhalb von 72 h rund 6 % der oral verabreichten TEHTM‑Dosis im Urin als Metaboliten wiedergefunden, wobei 2‑MEHTM den Hauptmetaboliten darstellte, gefolgt von 5cx‑1‑MEPTM, 5OH‑1‑MEHTM, 5OH‑2‑MEHTM und 1‑MEHTM, welche somit auch als Biomarker für ein Human­biomonitoring von TEHTM empfohlen werden können. Daher erfolgte in der hier beschriebenen Methode eine Eingrenzung der Metaboliten der ursprünglichen Methode von Höllerer et al. (2018 a) auf diese sechs Metaboliten (Kuhlmann et al. 2021).

## 3 Grundlage des Verfahrens

Das hier beschriebene Verfahren dient der Erfassung von sechs spezifischen Metaboliten des Weichmachers TEHTM in Urin. Bei den Analyten handelt es sich einerseits um zwei Monoester-Isomere als primäre Hydrolyseprodukte des TEHTM (1‑MEHTM und 2‑MEHTM), andererseits um die oxidativ gebildeten sekundären Derivate (5OH‑1‑MEHTM, 5OH‑2‑MEHTM, 5cx‑1‑MEPTM und 5cx‑2‑MEPTM). Die Bestimmung erfolgt nach enzymatischer Hydrolyse der Urinprobe sowie Anreicherung der Analyten mittels online‑SPE. Durch integrierte automatische Säulenschaltung werden die Analyten im Backflush-Mode auf die analytische Säule überführt, auf dieser flüssigkeitschromatographisch aufgetrennt und mittels Tandem-Massenspektrometrie quantifiziert. Die Kalibrierung erfolgt mit Kalibrierstandards, die in Poolurin angesetzt und in der gleichen Weise aufgearbeitet werden, wie die zu analysierenden Proben. Als interne Standards (ISTDs) werden den Urinproben folgende isotopenmarkierte Substanzen zugesetzt: D_5_‑1‑MEHTM, D_5_‑2‑MEHTM, D_5_‑5OH‑1‑MEHTM, D_5_‑5cx‑1‑MEPTM und D_5_‑5cx‑2‑MEPTM.

## 4 Geräte, Chemikalien und Lösungen

### 4.1 Geräte

- UPLC‑Anlage (z. B. ACQUITY UPLC H‑Class System, Waters GmbH, Eschborn) mit einer quaternären Pumpe (z. B. ACQ H‑Class QSM Plus, Waters GmbH, Eschborn), einer binären Pumpe (z. B. UPLC Binary SOL MGR, Waters GmbH, Eschborn), einem Autosampler (z. B. ACQ H‑Class FTN‑H Plus, Waters GmbH, Eschborn) sowie einem Säulenmanager (z. B. ACQUITY UPLC CM‑A, Waters GmbH, Eschborn)
- Triple-Quadrupol-Massenspektrometer (z. B. Xevo TQ‑XS, Waters GmbH, Eschborn)
- Software für Datenauswertung (z. B. MassLynx 4.2, TargetLynx XS, Waters GmbH, Eschborn)
- Anreicherungssäule (z. B. Nr. 186005233, XBridge^®^ BEH C8 Direct Connect HP (30 mm × 2,1 mm, 10 μm), restricted access material (RAM)‑Phase, Waters GmbH, Eschborn)
- Vorsäule für LC‑Säule (z. B. Nr. 9309A0252, Raptor^TM^ Biphenyl mit EXP^®^ Direct Connect Vorsäulenkartuschenhalter (5 mm × 2,1 mm, 2,7 μm), Restek Corporation, Bellefonte, PA, USA)
- Analytische UPLC‑Säule (z. B. Nr. 9309212, Core-Shell Raptor^TM^ Biphenyl (100 mm × 2,1 mm, 1,8 μm), Restek Cor­poration, Bellefonte, PA, USA)
- In-Line-Filter (z. B. Nr. 205000343, ACQUITY Column In-Line-Filter Waters Critical Clean^TM^, Waters GmbH, Esch­born)
- Laborzentrifuge (z. B. Megafuge^TM^, Heraeus Holding GmbH, Hanau)
- Vortex-Schüttler (z. B. Vortex 2, IKA‑Werke GmbH & Co. KG, Staufen)
- Thermostatierbarer Trockenschrank (z. B. Memmert GmbH + Co. KG, Schwabach)
- 1,8‑ml-Gewindefläschchen, klar, mit 8‑mm-Schraubverschlüssen mit PTFE‑beschichteten Septen (z. B. Nr. 548‑0018 und Nr. 548‑0024, VWR International GmbH, Darmstadt)
- 2‑ml-Gewindefläschchen, zertifiziert für LC‑MS, klar, mit 12‑mm-Schraubverschlüssen mit vorgeschlitzten PFTE‑beschichteten Septen (z. B. Nr. 600000668CV, Waters GmbH, Eschborn)
- Mikroliterpipetten, mit variabler Volumeneinstellung zwischen 10 μl und 100 μl sowie zwischen 100 μl und 1000 μl (z. B. Eppendorf AG, Hamburg) mit passenden Pipettenspitzen (z. B. FinnTip^TM^, Thermo Fisher Scientific^TM^, Life Technologies GmbH, Darmstadt)
- Multipetten^TM^ (z. B. M4, Eppendorf AG, Hamburg)
- Verschiedene Messkolben, Messzylinder und Bechergläser (z. B. DURAN^®^, Schott AG, Mainz)
- HPLC‑Fließmittelflaschen, 1 l (z. B. Waters GmbH, Eschborn oder DURAN^®^, Schott AG, Mainz)
- Magnetrührer (z. B. RSM‑10HS, Phoenix Instrument GmbH, Garbsen)
- pH‑Meter (z. B. Mettler-Toledo GmbH, Gießen)
- Urinbecher (weichmacherfrei, z. B. aus Polypropylen, Sarstedt AG & Co. KG, Nümbrecht)

### 4.2 Chemikalien

Wenn nicht anders angegeben, sind alle genannten Chemikalien mindestens in p. a.‑Qualität zu verwenden.

- 1‑MEHTM (z. B. Nr. B190025, Toronto Research Chemicals Inc., Toronto, Kanada)
- 2‑MEHTM (z. B. Nr. B190020, Toronto Research Chemicals Inc., Toronto, Kanada)
- 5OH‑1‑MEHTM, 99 % (z. B. Auftragssynthese, Max-Planck-Institut für Biophysikalische Chemie, Fakultät für synthetische organische Chemie, Göttingen)
- 5OH‑2‑MEHTM, 95 % (z. B. Auftragssynthese, Max-Planck-Institut für Biophysikalische Chemie, Fakultät für synthetische organische Chemie, Göttingen)
- 5cx‑1‑MEPTM, 95 % (z. B. Auftragssynthese, Max-Planck-Institut für Biophysikalische Chemie, Fakultät für synthetische organische Chemie, Göttingen)
- 5cx‑2‑MEPTM, 95 % (z. B. Auftragssynthese, Max-Planck-Institut für Biophysikalische Chemie, Fakultät für synthetische organische Chemie, Göttingen)
- Mischung aus D_5_‑1‑MEHTM und D_5_‑2‑MEHTM (D_5_‑1‑MEHTM ∶ D_5_‑2‑MEHTM, 40 ∶ 60, w/w), 95 % (z. B. Auftrags­synthese, Max-Planck-Institut für Biophysikalische Chemie, Fakultät für synthetische organische Chemie, Göttingen)
- D_5_‑5OH‑1‑MEHTM, 95 % (z. B. Auftragssynthese, Max-Planck-Institut für Biophysikalische Chemie, Fakultät für synthetische organische Chemie, Göttingen)
- D_5_‑5cx‑1‑MEPTM, 95 % (z. B. Auftragssynthese, Max-Planck-Institut für Biophysikalische Chemie, Fakultät für synthetische organische Chemie, Göttingen)
- D_5_‑5cx‑2‑MEPTM, 97 % (z. B. Auftragssynthese, Max-Planck-Institut für Biophysikalische Chemie, Fakultät für synthetische organische Chemie, Göttingen)
- Acetonitril (z. B. Nr. 83640, HiPerSolv CHROMANORM^®^ für die LC‑MS, VWR International GmbH, Darmstadt)
- Ameisensäure (z. B. Nr. 84865, HiPerSolv CHROMANORM^®^ für die LC‑MS, VWR International GmbH, Darmstadt)
- Ammoniumacetat (z. B. Nr. 1.01116, Merck KGaA, Darmstadt)
- Essigsäure (100 %, Eisessig) (z. B. Nr. 1.00063, Merck KGaA, Darmstadt)
- Hochreines Wasser (z. B. Nr. 83645, HiPerSolv CHROMANORM^®^ für die LC‑MS, VWR International GmbH, Darmstadt)
- Methanol (z. B. Nr. 83638, HiPerSolv CHROMANORM^®^ für die LC‑MS, VWR International GmbH, Darmstadt)
- 2‑Propanol (z. B. Nr. 84881, HiPerSolv CHROMANORM^®^ für die LC‑MS, VWR International GmbH, Darmstadt)
- *β*‑Glucuronidase aus *Escherichia coli* K12 (z. B. Nr. 03707598001, Roche Diagnostics Deutschland GmbH, Mannheim)
- Argon 5.0 (z. B. Linde GmbH, Pullach)
- Stickstoff 5.0 (z. B. Linde GmbH, Pullach)
- Nativer Urin von Freiwilligen mit möglichst geringen Hintergrundgehalten an TEHTM-Metaboliten

### 4.3 Lösungen

- Eluent A1 (Methanol)
  Als Eluent A1 wird Methanol verwendet.
- Eluent B1 (Wasser ∶ Methanol ∶ Ameisensäure (80 ∶ 20 ∶ 0,1; V/V/V))
  In einem Messzylinder werden 800 ml hochreines Wasser und 200 ml Methanol getrennt voneinander abgemessen und in eine leere Fließmittelflasche überführt. Anschließend wird 1 ml Ameisensäure zugegeben. Die Fließmittelflasche wird verschlossen und gut geschüttelt.
- Eluent A2 (Wasser ∶ Ameisensäure (100 ∶ 0,1; V/V))
  In einem Messzylinder werden 1000 ml hochreines Wasser abgemessen und in eine leere Fließmittelflasche überführt. Anschließend wird 1 ml Ameisensäure zugegeben. Die Fließmittelflasche wird verschlossen und gut geschüttelt.
- Eluent B2 (Acetonitril ∶ Ameisensäure (100 ∶ 0,1; V/V))
  In einem Messzylinder werden 1000 ml Acetonitril abgemessen und in eine leere Fließmittelflasche überführt. Anschließend wird 1 ml Ameisensäure zugegeben. Die Fließmittelflasche wird verschlossen und gut geschüttelt.

Die Eluenten sind bei Raumtemperatur mindestens drei Monate stabil.

- Ammoniumacetatpuffer (1 mol/l, pH 6,5)
  In einen 250‑ml-Becherglas werden genau 19,27 g Ammoniumacetat eingewogen und in etwa 200 ml hochreinem Wasser unter Rühren gelöst. Anschließend wird der pH‑Wert durch Zugabe von Eisessig auf pH 6,5 eingestellt. Die Lösung wird in einen 250‑ml-Messkolben überführt und dieser mit hochreinem Wasser bis zur Markierung aufgefüllt.

Die Lösung ist im Kühlschrank bei 4 °C mindestens drei Monate stabil.

### 4.4 Interne Standards (ISTDs)

- ISTD‑Stammlösungen (1000 mg/l)
  10 mg der Mischung aus D_5_‑1‑MEHTM und D_5_‑2‑MEHTM sowie je 10 mg D_5_‑5OH‑1‑MEHTM, D_5_‑5cx‑1‑MEPTM und D_5_‑5cx‑2‑MEPTM werden in je einen 10‑ml-Messkolben genau eingewogen und in Methanol gelöst. Die Kolben werden anschließend mit Methanol bis zur Markierung aufgefüllt.
- ISTD‑Arbeitslösung
  In einem 20‑ml-Messkolben werden etwa 5 ml Methanol vorgelegt. Anschließend werden 50 μl der Stammlösung von D_5_‑1‑MEHTM und D_5_‑2‑MEHTM sowie jeweils 20 μl der Stammlösungen von D_5_‑5OH‑1‑MEHTM, D_5_‑5cx‑1‑MEPTM und D_5_‑5cx‑2‑MEPTM zugegeben. Der Kolben wird anschließend mit Methanol bis zur Markierung aufgefüllt.
- ISTD‑Dotierlösung
  Von der ISTD‑Arbeitslösung werden 5 ml in einem 50‑ml-Messkolben mit Methanol verdünnt. Diese ISTD-Dotier­lösung enthält 100 μg D_5_‑1‑MEHTM/l, 150 μg D_5_‑2‑MEHTM/l und je 100 μg D_5_‑5OH‑1‑MEHTM, D_5_‑5cx‑1‑MEPTM und D_5_‑5cx‑2‑MEPTM/l.

Die Stammlösungen sowie die Arbeits- und die Dotierlösung werden bei −20 °C gelagert und sind unter diesen Bedin­gungen mindestens sechs Monate stabil.

### 4.5 Kalibrierstandards

- Stammlösungen (1000 mg/l)
  Es wird mit methanolischen Stammlösungen gearbeitet, deren jeweilige Analytkonzentration bei 1000 mg/l liegt.
- Dotierlösung I (100 μg/l)
  In einem 200‑ml-Messkolben werden etwa 50 ml hochreines Wasser vorgelegt. Anschließend werden jeweils 20 μl der einzelnen Stammlösungen zugegeben. Der Kolben wird mit hochreinem Wasser bis zur Markierung aufgefüllt.
- Dotierlösung II (10 μg/l)
  In einem 20‑ml-Messkolben werden etwa 5 ml hochreines Wasser vorgelegt und anschließend werden 2 ml der Dotierlösung I zugegeben. Der Kolben wird mit hochreinem Wasser bis zur Markierung aufgefüllt.

Die Stamm- und Dotierlösungen werden bei −20 °C gelagert und sind unter diesen Bedingungen mindestens sechs Monate stabil.

Für die Kalibrierung wird Poolurin von beruflich nicht gegen Weichmacher belasteten Personen verwendet. Eventuelle Hintergrundgehalte der jeweiligen Analyten in Urin werden subtrahiert. Für die Herstellung der Kalibrierstandards wird Urin nach dem in Tabelle 1 dargestellten Pipettierschema mit den Dotierlösungen versetzt. Als Leerwert wird der verwendete Poolurin mitgeführt. Die Kalibrierstandards werden für jede Analysenserie frisch hergestellt.

**Tab.1:** Pipettierschema zur Herstellung der Kalibrierstandards für die Bestimmung von TEHTM‑Metaboliten in Urin

**Kalibrierstandard**	**Dotierlösung I** **[μl]**	**Dotierlösung II** **[μl]**	**Poolurin** **[μl]**	Analytkonzentration [μg/l]
0	–	–	1000	0,0
1	–	10	990	0,1
2	–	20	980	0,2
3	–	50	950	0,5
4	10	–	990	1,0
5	20	–	980	2,0
6	50	–	950	5,0

## 5 Probenahme und Probenaufbereitung

### 5.1 Probenahme

Die Urinproben werden in verschließbaren Kunststoffbehältern (weichmacherfrei, z. B. aus Polypropylen) gesammelt und bei −20 °C eingefroren. So gelagert ist der Urin mindestens ein Jahr stabil.

### 5.2 Probenvorbereitung

Vor der Analyse werden die Proben bei Raumtemperatur aufgetaut und gut durchmischt. Ein Aliquot von jeweils 1 ml Urin wird in ein 1,8‑ml-Gewindefläschchen überführt, anschließend werden 200 μl Ammoniumacetatpuffer, 50 μl der ISTD-Dotierlösung sowie 10 μl der *β*‑Glucuronidase-Lösung zugegeben. Die Proben werden auf einem Vortexmischer gut durchmischt und für 2 h bei 37 °C im Trockenschrank inkubiert. Anschließend werden die Proben erneut gut gemischt und schließlich zur Unterbrechung der Hydrolyse bei −20 °C gelagert, bis sie gefroren sind (mind. 1 h, am besten über Nacht).

Danach werden die Proben aufgetaut und bei 3000 × *g* für 10 min zentrifugiert. Die Überstände werden unter Verwendung schmaler Pipettenspitzen (z. B. FinnTip^TM^, Thermo Fisher Scientific^TM^, Life Technologies GmbH, Darmstadt) in neue, gerätetaugliche Gewindefläschchen (2‑ml-Gewindefläschchen von Waters GmbH, Eschborn) überführt, welche anschließend verschlossen werden. Zur Analyse werden jeweils 30 μl der Proben in das LC‑MS/MS-System injiziert.

## 6 Instrumentelle Arbeitsbedingungen

**Abb.2:**
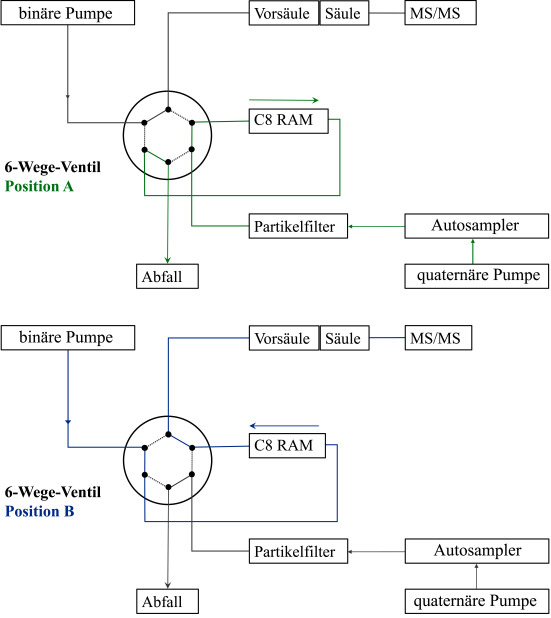
Skizze des Aufbaus der LC‑MS/MS-Apparatur mit Illustration der Schaltpositionen A (grün) und B (blau) des 6‑Wege-Ventils

Die analytische Messung erfolgt an einer Gerätekombination bestehend aus einer UPLC‑Anlage, einem 10‑Wege-Schaltventil sowie einem Tandem-Massenspektrometer. Für die Probenaufbereitung und Trennung werden zwei HPLC‑Pumpen benötigt (quaternäre Pumpe P1 und binäre Pumpe P2). Die zwei chromatographischen Säulen (RAM‑Phase und analytische Säule) werden über ein 6‑Wege-Ventil (sechs Wege des 10‑Wege-Schaltventils werden genutzt) miteinander gekoppelt (siehe Abbildung 2).

### 6.1 Flüssigkeitschromatographie

**Table:** 

Anreicherungssäule:	XBridge^®^ BEH C8 Direct Connect HP (30 mm × 2,1 mm, 10 μm)	
Vorsäule:	Raptor^TM^ Biphenyl (5 mm × 2,1 mm, 2,7 μm)	
Analytische Säule:	Core-Shell Raptor^TM^ Biphenyl (100 mm × 2,1 mm, 1,8 μm)	
Trennprinzip:	Umkehrphase (*reversed phase*)	
Mobile Phase der quaternären Pumpe P1:	Eluent A1:	Methanol
	Eluent B1:	Wasser ∶ Methanol ∶ Ameisensäure (80 ∶ 20 ∶ 0,1; V/V/V)
Mobile Phase der binären Pumpe P2:	Eluent A2:	Wasser ∶ Ameisensäure (100 ∶ 0,1; V/V)
	Eluent B2:	Acetonitril ∶ Ameisensäure (100 ∶ 0,1; V/V)
Flussrate der quaternären Pumpe P1:	0,5 ml/min; siehe Tabelle 2	
Flussrate der binären Pumpe P2:	0,3 ml/min; siehe Tabelle 3	
Schaltprogramm des Schaltventils:	siehe Tabelle 4	

**Tab.2:** Gradientenprogramm der quaternären Pumpe P1 zur online-Anreicherung der Analyten auf der RAM‑Phase

**Zeit** **[min]**	**Flussrate** **[ml/min]**	**Eluent A1** **[%]**	**Eluent B1** **[%]**
0	0,5	0	100
6,0	0,5	0	100
6,1	0,5	100	0
14,0	0,5	100	0
14,5	0,5	50	50
15,0	0,5	0	100
18,0	0,5	0	100

**Tab.3:** Gradientenprogramm der binären Pumpe P2 zur chromatographischen Trennung der Analyten

**Zeit** **[min]**	**Flussrate** **[ml/min]**	**Eluent A2** **[%]**	**Eluent B2** **[%]**
0	0,3	75	25
2,0	0,3	75	25
3,0	0,3	70	30
8,0	0,3	70	30
9,0	0,3	45	55
11,0	0,3	45	55
12,0	0,3	10	90
15,0	0,3	10	90
16,0	0,3	75	25
18,0	0,3	75	25

**Tab.4:** Schaltprogramm des 6‑Wege-Ventils (siehe auch Abbildung 2)

**Zeit** **[min]**	**Schalt­position**	**Beschreibung**
0–4,0	A	Anreicherung der Analyten auf der RAM‑Phase über Pumpe P1, Equilibrierung der analytischen Säule
4,0–6,0	B	Überführung der Analyten auf die analytische Säule über Pumpe P2, chromatographische Trennung der Analyten
6,0–11,0	A	Rekonditionierung der RAM‑Phase über Pumpe P1, chromatographische Trennung der Analyten über Pumpe P2
11,0–14,0	B	Spülen der RAM‑Phase auf die analytische Säule über Pumpe P2, chromatographische Trennung der Analyten
14,0–18,0	A	Rekonditionierung der RAM‑Phase über Pumpe P1 und der analytischen Säule über Pumpe P2

Es empfiehlt sich, vor die RAM‑Phase einen Partikelfilter einzufügen, um Verstopfungen der RAM‑Phase zu vermeiden. Alle anderen Parameter sind nach Herstellerangaben zu optimieren.

### 6.2 Tandem-Massenspektrometrie

**Table:** 

Ionisierung:	Elektrospray, negativ (ESI−)
Ionenspray-Spannung:	−4500 V
Kapillar-Spannung:	1 kV
Quellentemperatur:	150 °C
Desolvatisierungstemperatur:	500 °C
Dwelltime:	25 ms
Zerstäubergas:	Stickstoff, 700 kPa
Kollisionsgas:	Argon, 0,15 ml/min
Detektionsmodus:	*Multiple Reaction Monitoring* (MRM)

Die gerätespezifischen Parameter müssen vom Anwender individuell für das eingesetzte MS/MS‑System ermittelt und eingestellt werden. Die in diesem Abschnitt genannten gerätespezifischen Parameter sind für das während der Methodenentwicklung verwendete System bestimmt und optimiert worden.

## 7 Analytische Bestimmung

Zur analytischen Bestimmung der nach Abschnitt 5.2 aufgearbeiteten Urinproben wird ein Aliquot von 30 μl mittels Autosampler in das LC‑MS/MS-System injiziert. Nach online-Anreicherung und chromatographischer Trennung der Analyten werden diese anhand der Retentionszeiten sowie der charakteristischen Massenübergänge identifiziert (siehe Tabelle 5). Bei jeder Analysenserie werden hochreines Wasser als Leerwert sowie mindestens zwei Qualitätskontrollproben mitanalysiert. Isomere, die identische massenspektrometrische Zerfälle aufweisen, werden durch unterschiedliche Retentionszeiten voneinander unterschieden und identifiziert. Analyten mit ähnlicher Retentionszeit werden durch charakteristische Massenübergänge unterschieden.

**Tab.5:** Massenübergänge der Analyten und ISTDs, Retentionszeiten sowie weitere parameterspezifische Einstellungen

**Analyt oder ISTD**	**Vorläufer-Ion** **(*m/z*)**	**Produkt-Ion** **(*m/z*)**	**Cone-Voltage** **[V]**	**Kollisionsenergie** **[eV]**	**Retentionszeit** **[min]**
5OH‑2‑MEHTM	337	121^a)^	20	−22	6,3
		165	20	−18	
5cx‑1‑MEPTM	351	191^a)^	22	−12	6,3
		147	22	−22	
5cx‑2‑MEPTM	351	191^a)^	10	−12	6,7
		147	10	−24	
5OH‑1‑MEHTM	337	121^a)^	22	−24	6,7
		165	22	−18	
2‑MEHTM	321	277^a)^	22	−12	9,9
		233	22	−18	
1‑MEHTM	321	150^a)^	2	−14	10,1
		178	2	−16	
D_5_‑5cx‑1‑MEPTM	356	121	8	−28	6,3
D_5_‑5cx‑2‑MEPTM	356	191	14	−14	6,7
D_5_‑5OH‑1‑MEHTM	342	165	10	−18	6,7
D_5_‑2‑MEHTM	326	238	12	−16	9,9
D_5_‑1‑MEHTM	326	150	12	−16	10,1

a) Quantifier

**Abb.3:**
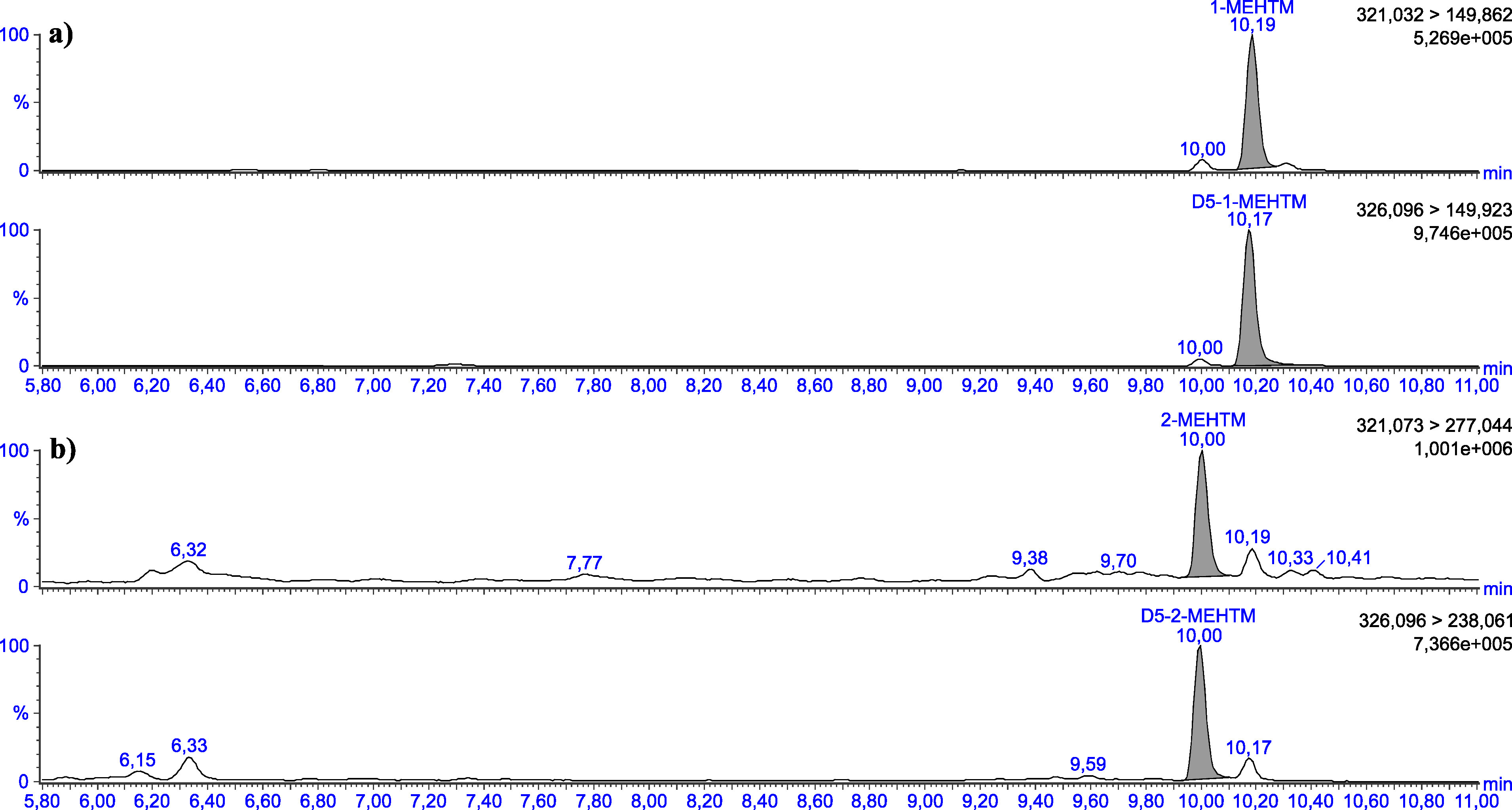
Chromatogramme einer mit 2 μg 1‑MEHTM (a) sowie mit 2 μg 2‑MEHTM (b) pro Liter dotierten Urinprobe

Die in Tabelle 5 angegebenen Retentionszeiten können nur als Anhaltspunkt dienen. Der Anwender dieser Methode hat sich selbst von der Trennleistung der von ihm verwendeten analytischen Säule und dem daraus resultierenden Retentionsverhalten der Substanzen zu überzeugen. Beispielhafte Chromatogramme sind in den Abbildungen 3 bis 8 gezeigt.

**Abb.4:**
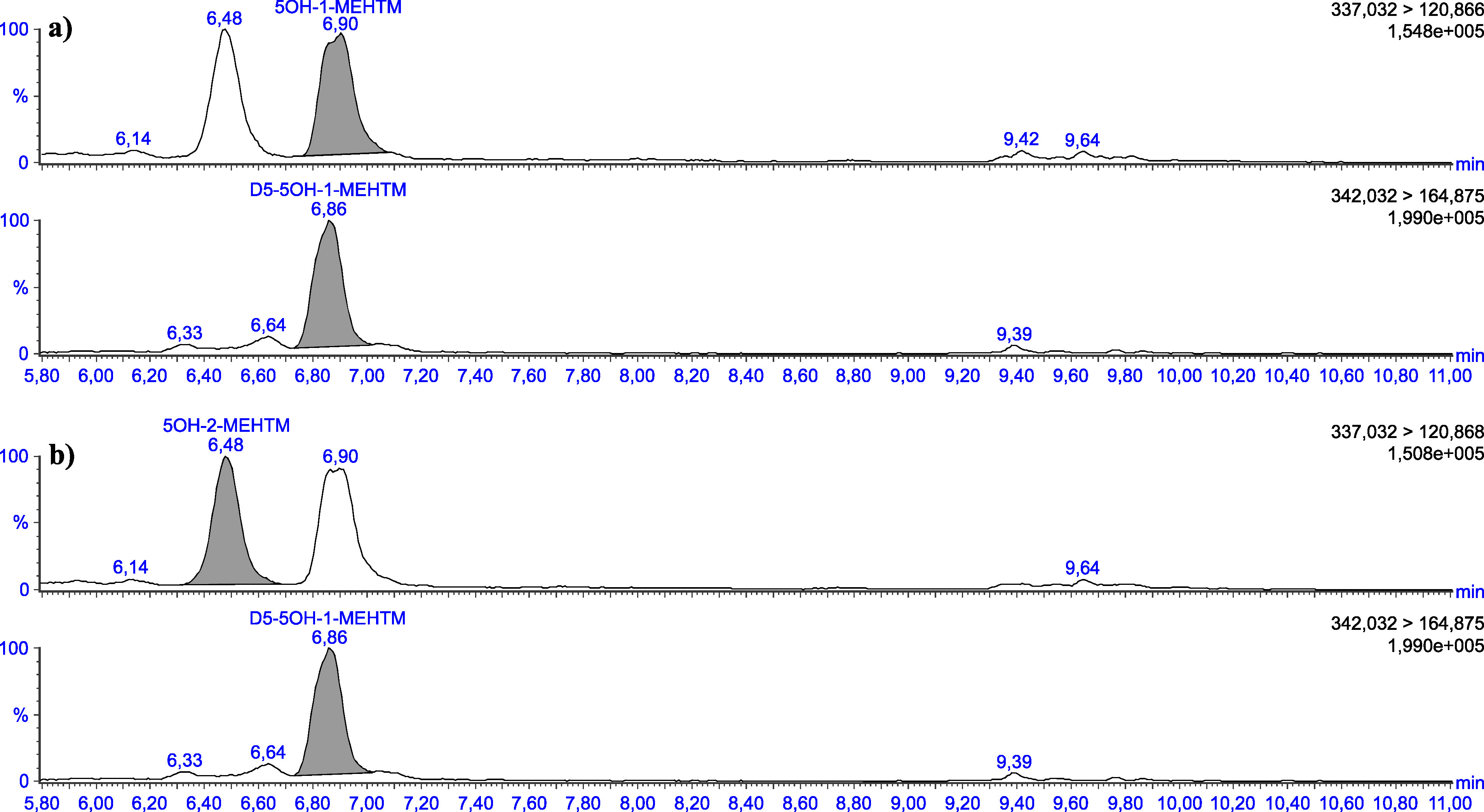
Chromatogramme einer mit 2 μg 5OH‑1‑MEHTM (a) sowie mit 2 μg 5OH‑2‑MEHTM (b) pro Liter dotierten Urinprobe

**Abb.5:**
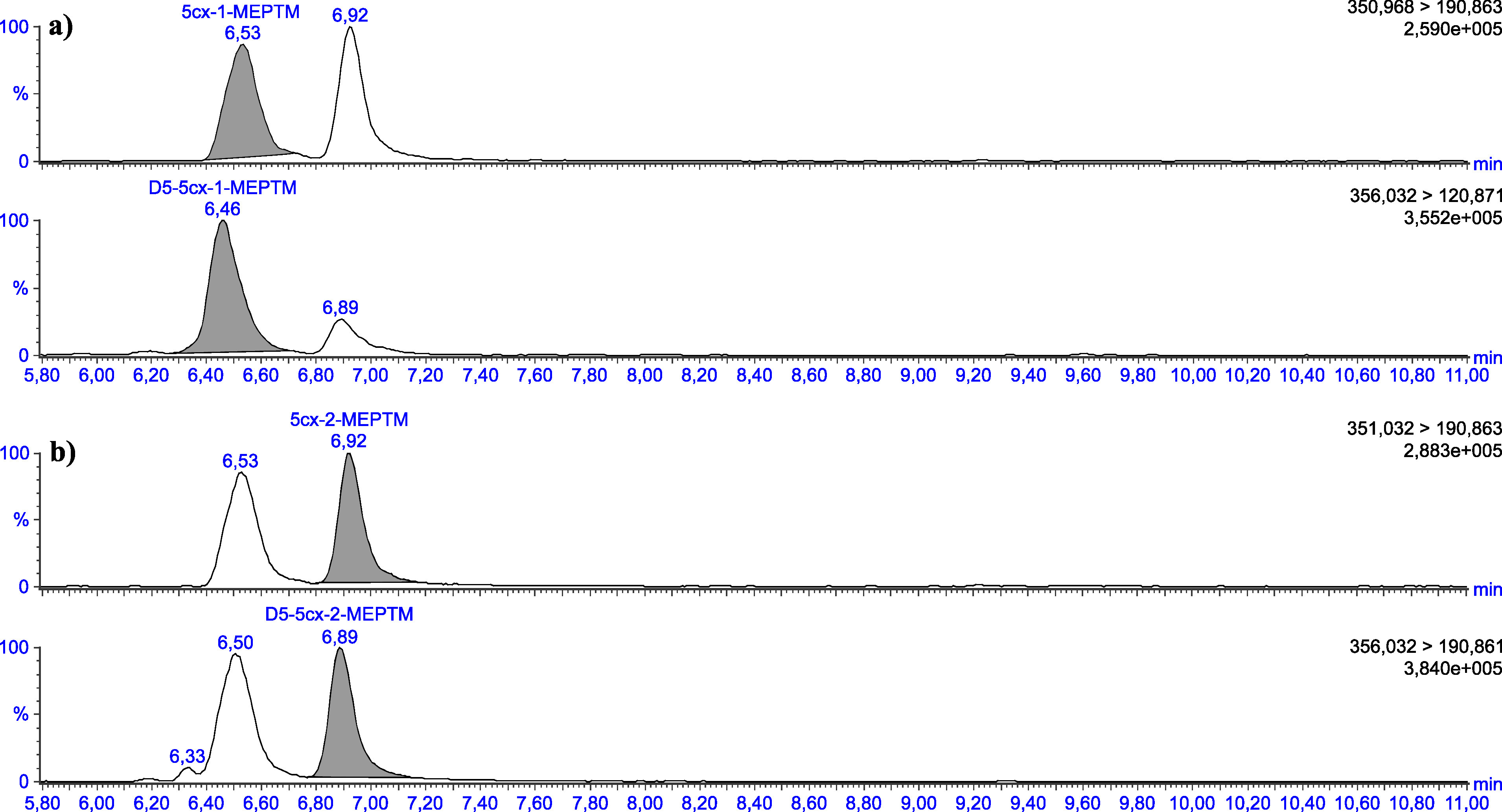
Chromatogramme einer mit 2 μg 5cx‑1‑MEPTM (a) sowie mit 2 μg 5cx‑2‑MEPTM (b) pro Liter dotierten Urinprobe

**Abb.6:**
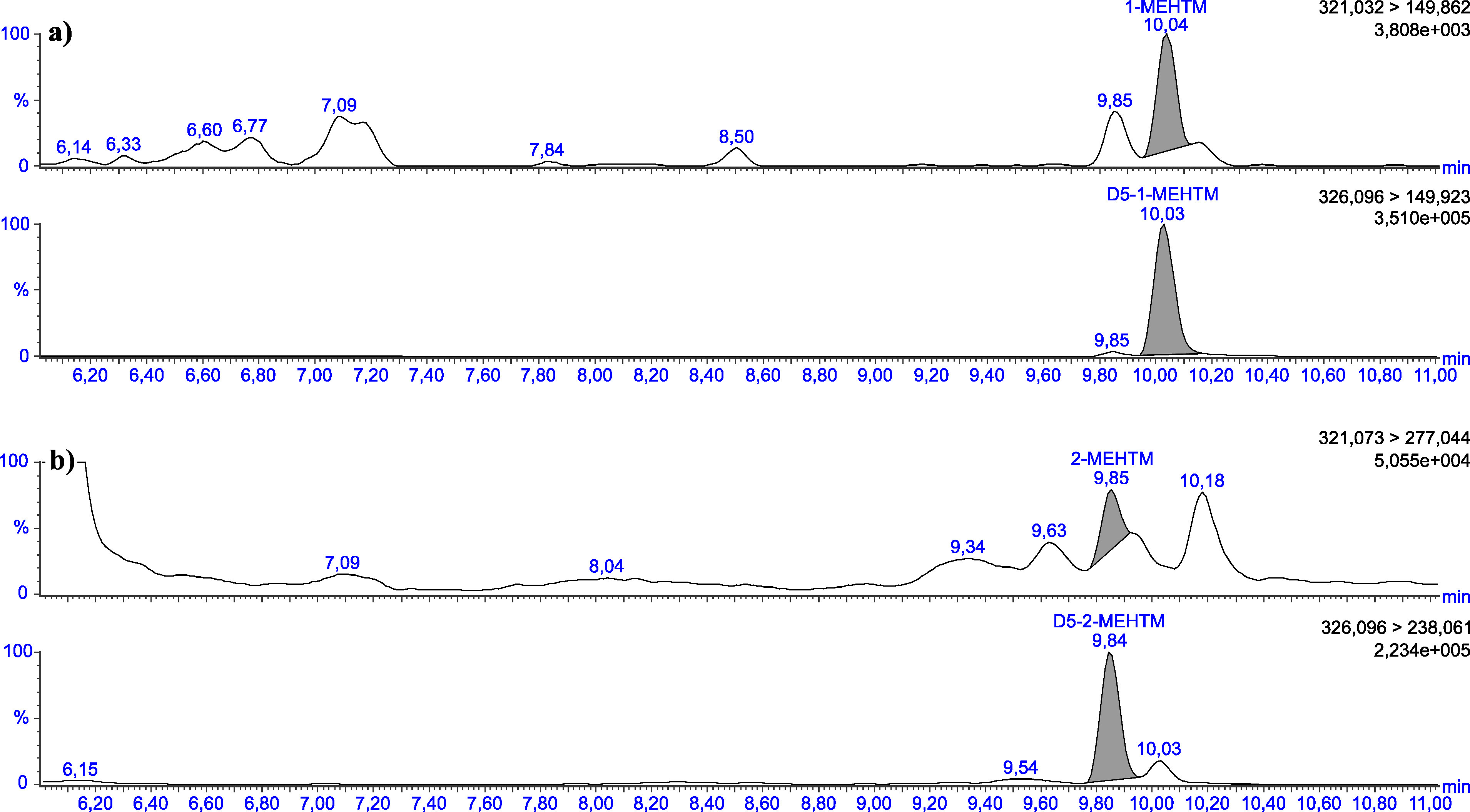
Chromatogramme einer nativen Urinprobe mit einer Konzentration von 0,03 μg 1‑MEHTM/l (a) sowie 0,26 μg 2‑MEHTM/l (b)

**Abb.7:**
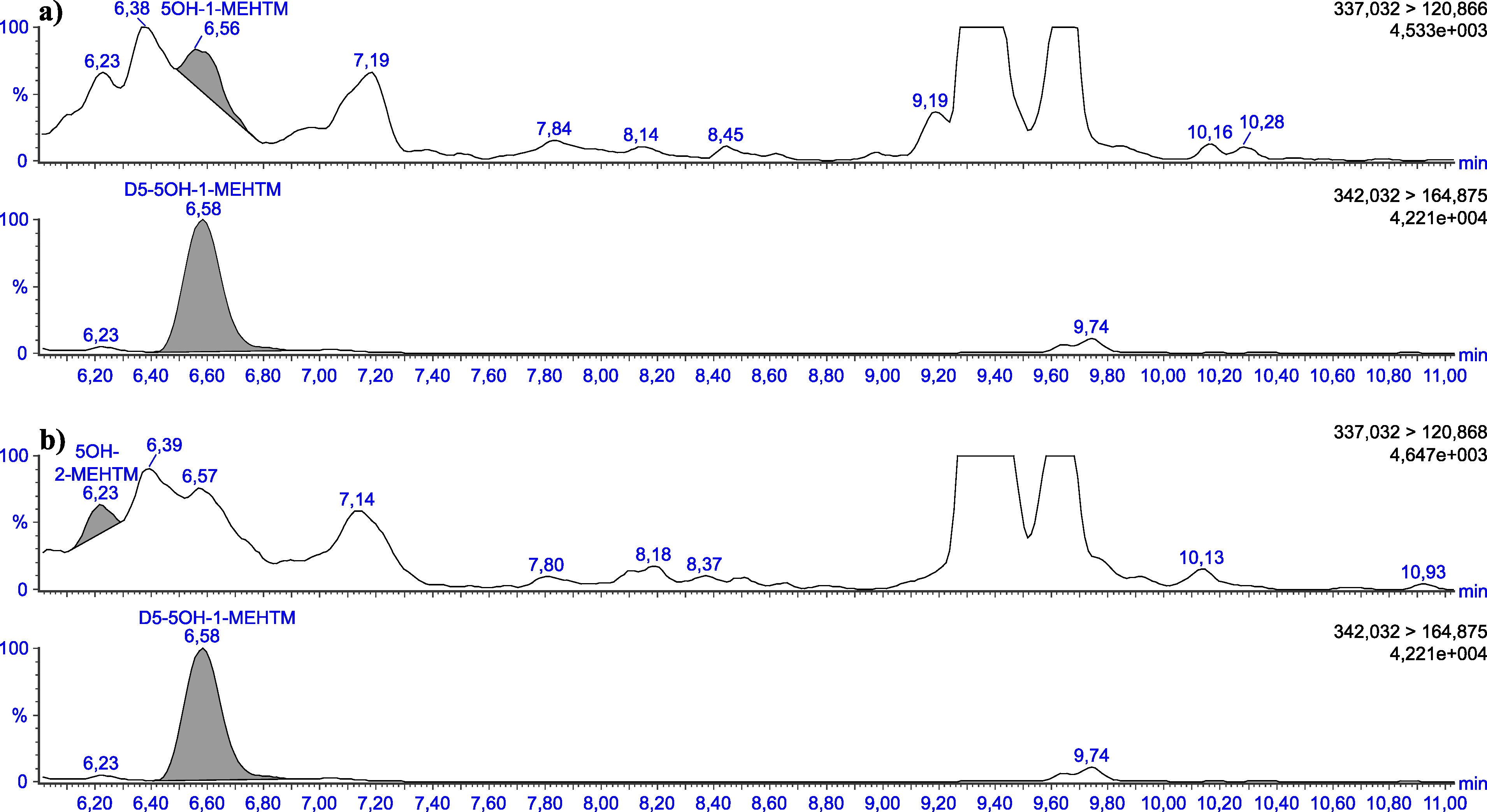
Chromatogramme einer nativen Urinprobe mit einer Konzentration von 0,11 μg 5OH‑1‑MEHTM/l (a) sowie 0,06 μg 5OH‑2‑MEHTM/l (b)

**Abb.8:**
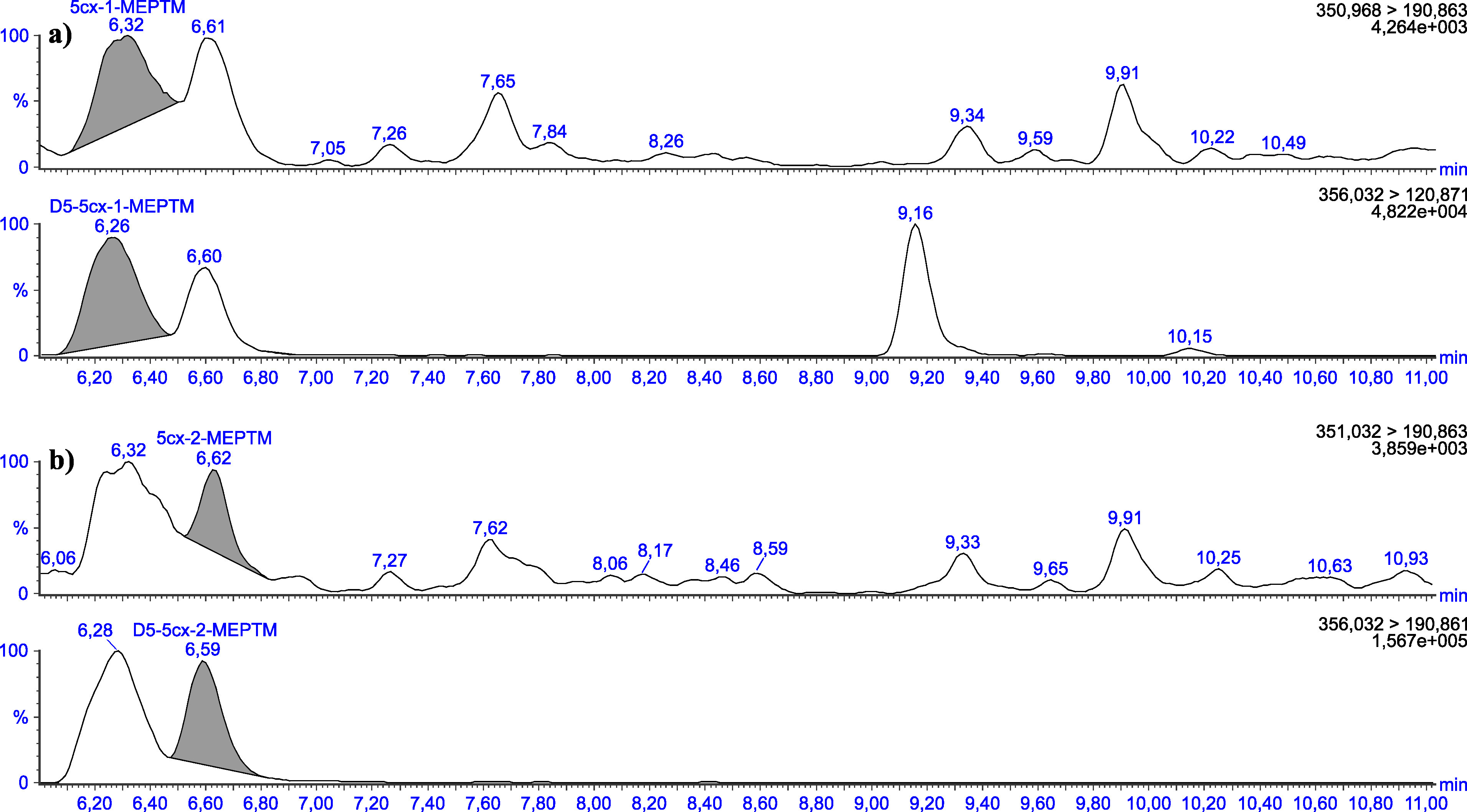
Chromatogramme einer nativen Urinprobe mit einer Konzentration von 0,19 μg 5cx‑1‑MEPTM/l (a) sowie 0,04 μg 5cx‑2‑MEPTM/l (b)

## 8 Kalibrierung

**Abb.9:**
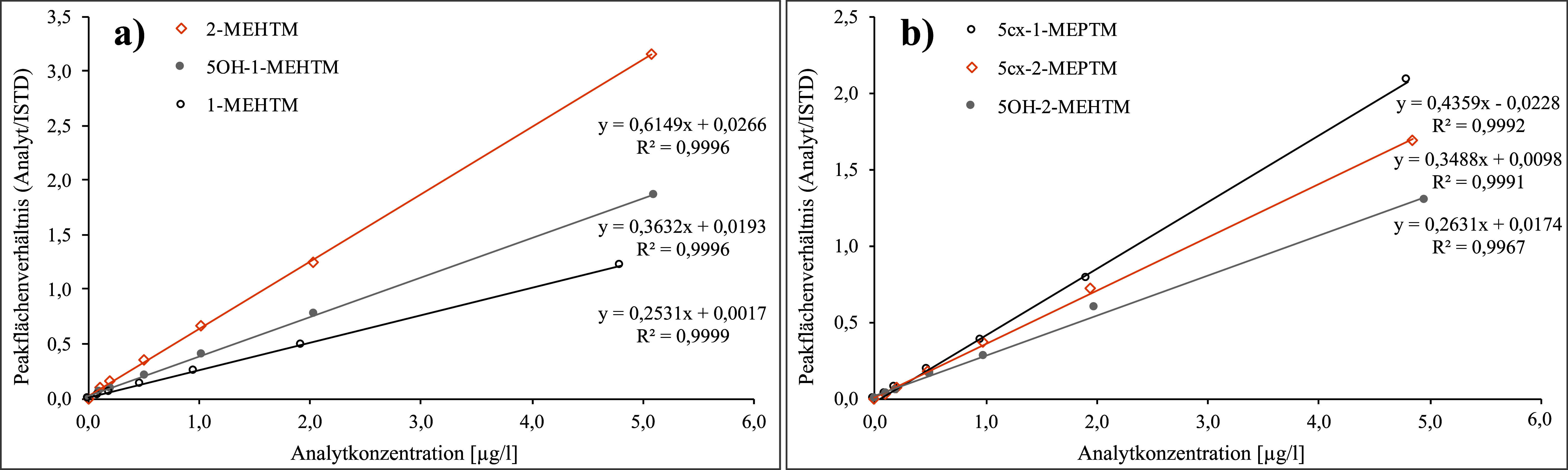
Beispielhafte Kalibriergeraden zur Bestimmung der TEHTM-Metaboliten in Urin

Die Kalibrierstandards (siehe Abschnitt 4.5) werden analog zu den Urinproben gemäß Abschnitt 5 aufgearbeitet und entsprechend den Abschnitten 6 und 7 analysiert. Die Kalibriergeraden werden erstellt, indem die Quotienten aus der Peakfläche des Analyten und des zugehörigen ISTDs gegen die dotierte Konzentration der jeweiligen Kalibrierstandards aufgetragen werden. Für den Analyten 5OH‑2‑MEHTM wird D_5_‑5OH‑1‑MEHTM als ISTD verwendet. Für alle anderen Analyten werden strukturidentische isotopenmarkierte Standards eingesetzt. Beispielhafte Kalibriergeraden sind in Abbildung 9 gegeben.

## 9 Berechnung der Analysenergebnisse

Der Analytgehalt einer Probe wird bestimmt, indem der Quotient aus der Peakfläche des Analyten und der Peakfläche des dazugehörigen ISTDs gebildet und in die entsprechende Kalibrierfunktion eingesetzt wird. Man erhält die Analyt­konzentration in μg/l. Eventuell auftretende Reagenzienleerwerte werden durch Subtraktion berücksichtigt.

## 10 Standardisierung der Messergebnisse und Qualitätssicherung

Zur Sicherung der Qualität der Analysenergebnisse wird gemäß den Richtlinien der Bundesärztekammer und den Angaben in dem von der Kommission veröffentlichten allgemeinen Kapitel verfahren (Bader et al. 2010; Bundesärztekammer 2023).

Zur Präzisionskontrolle werden mit jeder Analysenserie mindestens zwei Urinproben, die bekannte Analyt­kon­zentra­tionen aufweisen, als Qualitätskontrollen mitgeführt. Da ein derartiges Qualitätskontrollmaterial kommerziell nicht erhältlich ist, muss es selbst im Labor hergestellt werden. Dazu wird Poolurin mit einer definierten Menge der Analyten dotiert, aliquotiert und bei −20 °C gelagert. Für die Validierung dieser Methode wurden zwei Kontrollmaterialien hergestellt: das Kontrollmaterial Q_low_ enthielt etwa 0,25 μg/l der jeweiligen Analyten, während das Kontrollmaterial Q_high_ etwa 1,25 μg/l enthielt.

Zusätzlich wird bei jeder Analysenserie ein Reagenzienleerwert, bestehend aus 1000 μl hochreinem Wasser, mitgemessen. Der Reagenzienleerwert wird analog zu den Urinproben aufgearbeitet und analysiert.

## 11 Beurteilung des Verfahrens

Die Zuverlässigkeit des Verfahrens wurde durch eine umfassende Validierung sowie durch Nachstellung und Prüfung der Methode in einem zweiten, unabhängigen Labor bestätigt.

### 11.1 Präzision

Zur Bestimmung der Präzision in der Serie wurde das nach Abschnitt 10 hergestellte Qualitätskontrollmaterial mehrfach parallel aufgearbeitet und vermessen. Bei der zehnfachen Bestimmung dieser Urinproben ergaben sich die in Tabelle 6 dokumentierten Präzisionsdaten.

**Tab.6:** Präzision in der Serie für die Bestimmung der TEHTM‑Metaboliten in Urin (n = 10)

Analyt	**Dotierte Konzentration** **[μg/l]**	**Standardabweichung (rel.) *s_w_*** **[%]**	Streubereich ***u*** **[%]**
1‑MEHTM	0,24	3,0	6,8
	1,20	6,3	14,3
2‑MEHTM	0,25	4,3	9,7
	1,27	6,3	14,3
5OH‑1‑MEHTM	0,26	5,0	11,3
	1,28	4,0	9,0
5OH‑2‑MEHTM	0,25	8,1	18,3
	1,24	5,5	12,4
5cx‑1‑MEPTM	0,24	2,4	5,4
	1,20	6,0	13,6
5cx‑2‑MEPTM	0,24	4,8	10,9
	1,21	6,0	13,6

Zur Ermittlung der Präzision von Tag zu Tag wurden die Qualitätskontrollproben an acht unterschiedlichen Tagen aufgearbeitet und analysiert. Daraus ergaben sich die in Tabelle 7 dokumentierten Präzisionsdaten.

**Tab.7:** Präzision von Tag zu Tag für die Bestimmung der TEHTM‑Metaboliten in Urin (n = 8)

**Analyt**	**Dotierte Konzentration** **[μg/l]**	Standardabweichung (rel.) ***s_w_*** **[%]**	Streubereich ***u*** **[%]**
1‑MEHTM	0,24	5,2	12,3
	1,20	6,6	15,9
2‑MEHTM	0,25	4,0	9,5
	1,27	7,3	17,3
5OH‑1‑MEHTM	0,26	5,0	11,8
	1,28	3,7	8,8
5OH‑2‑MEHTM	0,25	5,9	14,0
	1,24	2,7	6,3
5cx‑1‑MEPTM	0,24	6,0	14,2
	1,20	4,8	11,4
5cx‑2‑MEPTM	0,24	4,8	11,4
	1,21	5,6	13,2

### 11.2 Richtigkeit

Die relative Wiederfindung der Analyten wurde aus den Daten der Präzision von Tag zu Tag berechnet. Es ergaben sich die in Tabelle 8 aufgeführten relativen Wiederfindungen.

**Tab.8:** Relative Wiederfindung für die Bestimmung der TEHTM‑Metaboliten in Urin (n = 8)

**Analyt**	**Dotierte Konzentration** **[μg/l]**	Mittlere Wiederfindung (rel.) ***r*** **[%]**	**Bereich** **[%]**
1‑MEHTM	0.24	100	89.6–106
	1.20	99.0	93.7–113
2‑MEHTM	0.25	104	99.6–109
	1.27	96.8	90.1–111
5OH‑1‑MEHTM	0.26	98.9	91.4–107
	1.28	99.0	95.9–107
5OH‑2‑MEHTM	0.25	104	93.1–109
	1.24	109	104–112
5cx‑1‑MEPTM	0.24	105	95.9–115
	1.20	109	103–118
5cx‑2‑MEPTM	0.24	99.4	91.5–108
	1.21	100	88.9–109

### 11.3 Nachweis- und Bestimmungsgrenzen

Die Nachweis- und Bestimmungsgrenzen wurden mit der Kalibriergeradenmethode nach DIN 32645 bestimmt (DIN 2008). Die äquidistanten zehn-Punkt-Kalibriergeraden überspannten den Konzentrationsbereich von 0,05–0,50 μg/l. In Tabelle 9 sind die ermittelten Nachweis- und Bestimmungsgrenzen für die einzelnen Analyten aufgeführt.

**Tab.9:** Nachweis- und Bestimmungsgrenzen für die Bestimmung der TEHTM‑Metaboliten in Urin

**Analyt**	**Nachweisgrenze ** **[μg/l]**	**Bestimmungsgrenze ** **[μg/l]**
1‑MEHTM	0,01	0,04
2‑MEHTM	0,02	0,07
5OH‑1‑MEHTM	0,02	0,07
5OH‑2‑MEHTM	0,04	0,12
5cx‑1‑MEPTM	0,01	0,05
5cx‑2‑MEPTM	0,01	0,04

### 11.4 Stabilität der Analyten beim Einfrieren/Auftauen sowie bei Raumtemperatur

Um die Stabilität der Analyten während der Probenlagerung zu prüfen, wurden Lagerungsversuche durchgeführt. Dazu wurde Urin mit den Analyten in einer Konzentration von 80 µg/l dotiert und drei Gefrier-Tau-Zyklen unterzogen. Alternativ wurden die dotierten Proben vier Tage bei Raumtemperatur im Dunkeln gelagert. Die gelagerten Proben wurden zusammen mit frisch dotierten Proben in einem Analysenlauf gemessen, wobei diese jeweils vor und nach der jeweiligen gelagerten Probe gemessen wurden. Die Ergebnisse dieser Messungen sind in Tabelle 10 gezeigt. 

Nach viertägiger Lagerung bei Raumtemperatur ergaben sich für alle Analyten sehr gute Wiederfindungen, die zwischen 95 und 110 % lagen. Nach Abschluss der drei Gefrier-Tau-Zyklen war die Wiederfindung von 1-MEHTM und 2-MEHTM mit 87 bzw. 85 % akzeptabel. Für die übrigen Analyten waren die Wiederfindungen nach drei Gefrier-Tau-Zyklen sehr gut. Es ist anzumerken, dass die für die Stabilitätstests gewählte Analytkonzentration im Bereich möglicher hoher beruflicher Belastungen lag. Sofern nötig, sollten Anwender der Methode die Lagerungsstabilität in einem niedrigeren Konzentrationsbereich überprüfen.

**Tab.10:** Stabilität der TEHTM‑Metaboliten in Urin nach Probenlagerung

**Analyt**	Mittlere Wiederfindung (rel.) ***r *** **[%]**	
	nach drei Gefrier-Tau-Zyklen	nach vier Tagen bei Raumtemperatur
1‑MEHTM	87,4	98,9
2‑MEHTM	85,4	101,0
5OH‑1‑MEHTM	91,4	94,6
5OH‑2‑MEHTM	106,5	110,3
5cx‑1‑MEPTM	109,4	99,4
5cx‑2‑MEPTM	103,9	98,3

### 11.5 Störeinflüsse

Die hier beschriebene Methode zur Bestimmung der TEHTM‑Metaboliten ermöglicht die zuverlässige Bestimmung dieser Parameter in Urin. Zum Erhalt der Methodenqualität sollten jedoch verschiedene Punkte Beachtung finden.

Die Analyten der Methode bestehen aus Gruppen von Isomeren, die dieselben massenspektrometrischen Frag­men­tierungen aufweisen. Diese sind a) die Gruppe der sekundären Carboxy‑Metaboliten (5cx‑1‑MEPTM und 5cx‑2‑MEPTM) mit den Massenfragmenten *m/z* 147 und *m/z* 191, b) die Gruppe der sekundären Hydroxy‑Metaboliten (5OH‑1‑MEHTM und 5OH‑2‑MEHTM) mit den Massenfragmenten *m/z* 121 und *m/z* 165 sowie c) die Gruppe der primären Monoester-Isomere (1‑MEHTM und 2‑MEHTM) mit den Massenfragmenten *m/z* 150, *m/z* 178, *m/z* 277 und *m/z* 233. Für die eindeutige, selektive Identifizierung der jeweiligen Isomere ist eine chromatographische Basislinientrennung der Peaks erforderlich. Dies wurde in der vorliegenden Methode durch Verwendung einer Core-Shell-Biphenyl-Säule mit geringen Dimensionen sowie durch Optimierung des Laufmittelgradienten erreicht. Nach etwa 100 Injektionen sollten sowohl die Vorsäule als auch der Partikelfilter erneuert werden.

In einigen Fällen wurde beobachtet, dass Matrixbestandteile des Urins die Auswertung der Analyten 5OH‑1‑MEHTM, 5OH‑2‑MEHTM und 5cx‑1‑MEPTM störten. Diese Störung konnte durch ausgiebiges Spülen des chromatographischen Systems mit organischen Lösungsmitteln verringert werden. Um Störungen durch die Urinmatrix zu vermeiden, sollten zudem ausreichend Leerinjektionen in die Messsequenz eingebaut werden, insbesondere nach Urinproben mit hohen Kreatininwerten.

Bei Analytkonzentrationen über 10 μg/l kann es zu Verschleppungen kommen. Sofern diese beobachtet werden, empfiehlt es sich, nur mit Konzentrationen im Bereich von 0–10 μg/l zu arbeiten.

## 12 Diskussion der Methode

Die Methode ermöglicht die zuverlässige Bestimmung von insgesamt sechs TEHTM-Metaboliten in Urin. Um alle Metaboliten eindeutig identifizieren zu können, werden die Carboxy‑, Hydroxy- sowie Monoester-Isomere, die jeweils die gleichen Fragmentierungsreaktionen aufweisen, auf der chromatographischen Säule basisliniengetrennt.

Wie bereits publizierte In‑vitro- und In‑vivo-Studien zeigen, kommt es im menschlichen Körper nach Resorption von TEHTM zunächst zu einer regioselektiven Esterspaltung, wodurch die Monoester-Isomere 1‑MEHTM und 2‑MEHTM entstehen, die auch die Hauptmetaboliten des TEHTM sind. Von den oxidativ gebildeten sekundären Folgeprodukten dieser Isomere stellen 5OH‑1‑MEHTM, 5OH‑2‑MEHTM und 5cx‑1‑MEPTM die in vivo quantitativ wichtigsten Meta­boliten dar (Höllerer et al. 2018 a).

Generell zeichnet sich die Methode durch eine einfache Probenaufarbeitung aus. Die erhobenen Validierungsdaten belegen, dass es sich um ein sensitives und zuverlässiges Verfahren handelt, das sehr gut geeignet ist, die innere Belastung von Personen zu erfassen, die gegen den Weichmacher TEHTM exponiert sind. Da TEHTM bislang vorwiegend für Medizinprodukte verwendet wird, stellt die Behandlung von Patienten unter Verwendung von Medizinprodukten, die TEHTM als Weichmacher enthalten, die wichtigste Expositionsquelle für diese Substanz dar. 

Die Exposition der Allgemeinbevölkerung ist dagegen als gering einzuschätzen. So war die Nachweishäufigkeit der mit dieser Methode bestimmbaren TEHTM-Metaboliten im Urin von Jugendlichen in Deutschland sehr gering (Murawski et al. 2021). Während 2‑MEHTM in elf der 439 untersuchten Urinproben in Konzentrationen oberhalb der Bestimmungsgrenze vorlag, konnte 5cx‑2‑MEPTM in keiner der Proben quantifiziert werden. Die übrigen Metaboliten konnten in 0,23–0,68 % der Urinproben bestimmt werden. Die Expositionsursache konnte bei den gegen TEHTM exponierten Personen nicht eindeutig geklärt werden.

**Verwendete Messgeräte** UPLC‑Anlage (ACQUITY UPLC H‑Class System, Waters GmbH, Eschborn) mit einer quaternären Pumpe (ACQ H‑Class QSM Plus, Waters GmbH, Eschborn), einer binären Pumpe (UPLC Binary SOL MGR, Waters GmbH, Eschborn), einem Autosampler (ACQ H‑Class FTN‑H Plus, Waters GmbH, Eschborn) sowie einem Säulenmanager (ACQUITY UPLC CM‑A, Waters GmbH, Eschborn); Triple-Quadrupol-Massenspektrometer (Xevo TQ‑XS, Waters GmbH, Eschborn)
